# Characterization of the Whole-Genome Sequence of a Beak and Feather Disease Virus Isolate from a Mallee Ringneck Parrot (*Barnardius zonarius barnardi*)

**DOI:** 10.1128/genomeA.00708-14

**Published:** 2014-07-24

**Authors:** Shubhagata Das, Subir Sarker, Jade K. Forwood, Seyed A. Ghorashi, Shane R. Raidal

**Affiliations:** a School of Animal and Veterinary Sciences, Charles Sturt University, Wagga Wagga, New South Wales, Australia; b School of Biomedical Sciences, Charles Sturt University, Wagga Wagga, New South Wales, Australia; c Graham Centre for Agricultural Innovation, Wagga Wagga, New South Wales, Australia

## Abstract

The complete genome sequence of beak and feather disease virus (BFDV) from a wild Australian Mallee ringneck parrot (*Barnardius zonarius barnardi*) was characterized. The genome consists of 1,995 nucleotides and encodes two major proteins in opposing directions. This is the first evidence of BFDV infectivity and the first complete genome sequence for this novel host.

## GENOME ANNOUNCEMENT

Psittacine beak and feather disease (PBFD) is a common viral infection that occurs in a wide variety of psittacine birds, globally affecting >60 different species (1–4). The disease is caused by one of the smallest and simplest viruses belonging to the family *Circoviridae*, beak and feather disease virus (BFDV), a nonenveloped icosahedral virus with an approximately 2.0-kb circular single-stranded DNA (ssDNA) genome. The genome typically encompasses two major bidirectionally transcribed open reading frames (ORFs) encoding replication-associated protein (Rep) and capsid protein (Cap), with a potential stem-loop structure located between them (5–7). Clinically, BFDV infection exhibits as either sudden death or a chronic progressive illness characterized by symmetrical feather loss and deformity in beak and claw (5, 6, 8). Under natural conditions, horizontal transmission is likely the major route of spread for this virus (9). Here, we characterize the complete BFDV genome isolated from a wild Mallee ringneck parrot (*Barnardius zonarius barnardi*) in Australia. Feather samples collected from a Mallee ringneck parrot (ID, 14-1195/001; year of sampling, 2014; global positioning system [GPS] location, 31.9558°S 141.4651°E) were used as a source of genomic DNA, for which extraction of DNA was performed according to established protocols (10–12). The whole-genome sequence was amplified using the primers and PCR conditions developed in previous studies (13, 14). Briefly, the optimized reaction mixture contained 3 µl extracted genomic DNA, 2.5 µl of 10-µl high-fidelity PCR buffer (Invitrogen), 1 µl of 25 µM (each) primers, 1 µl of 50 mM MgSO_4_, 4 µl of 1.25 mM dinucleoside triphosphates (dNTPs), 1 U Platinum *Taq* DNA polymerase high-fidelity (Invitrogen), and distilled water added for a final volume of 25 µl. The optimized PCR conditions were 95°C for 3 min, followed by 40 cycles of 95°C for 30 s, 57°C for 45 s, and 68°C for 2 min, and finally 68°C for 5 min. The amplified PCR products were TA cloned into pGEM-T vector (Promega, USA) and sequenced at the Australian Genome Research Facility (AGRF), Ltd. (Sydney, Australia). The sequenced contigs were assembled, and the entire BFDV genome was constructed using Geneious software (version 6.1.7).

The newly amplified BFDV genome (GenBank accession no. KJ866054) comprises 1,995 nucleotides (nt), with a G+C content of 53.3%. Similar to other BFDV genomes, the basic structure includes two major open reading frames (ORFs), ORF1 (870 nt) and ORF2 (708 nt), encoding a replication-associated protein (Rep) and the capsid protein (Cap), respectively. Phylogenetic analysis of this newly sequenced genome with all other BFDV genomes available on GenBank revealed the closest relationship (99% bootstrap support and 96% nucleotide sequence identity) with one of the Australian BFDV isolates obtained from a glossy black cockatoo (GenBank accession no. AF385408) (15). The overall nucleotide identity of this new isolate ranges from 92 to 96% compared with the BFDV genomes available on GenBank (16). This is the first report of a BFDV genome identification and characterization for this host species. This study documents the genomic characteristics and diversity of BFDV in a novel host (*B. zonarius barnardi*), which may facilitate further research on viral evolution and recombination events in this host species.

### Nucleotide sequence accession number.

The complete genome sequence of BFDV has been deposited at GenBank under the accession no. KJ866054.
